# Plasma EphA2 level is a superior biomarker to Del-1 for sepsis diagnosis and prognosis

**DOI:** 10.3389/fmed.2025.1505882

**Published:** 2025-01-24

**Authors:** Eun Hye Lee, Mi Hwa Shin, Se Hyun Kwak, Ji Soo Choi, Ah Young Leem, Su Hwan Lee, Kyung Soo Chung, Young Sam Kim, Sang-Guk Lee, Moo Suk Park

**Affiliations:** ^1^Division of Pulmonology, Allergy and Critical Care Medicine, Department of Internal Medicine, Yongin Severance Hospital, Yonsei University College of Medicine, Yongin, Republic of Korea; ^2^Division of Pulmonary and Critical Care Medicine, Department of Internal Medicine, Institute of Chest Diseases, Severance Hospital, Yonsei University College of Medicine, Seoul, Republic of Korea; ^3^Department of Laboratory Medicine, Severance Hospital, Yonsei University College of Medicine, Seoul, Republic of Korea

**Keywords:** EphA2, Del-1, sepsis, biomarker, diagnosis, prognosis

## Abstract

**Background:**

Sepsis, characterized by a dysregulated host response to infection, often leads to organ dysfunction, and vascular endothelial dysfunction plays a central role. The erythropoietin-producing hepatocellular carcinoma (Eph)A2 receptor is associated with increased vascular permeability; however, the developmental endothelial locus-1 (Del-1), has contrasting effects on endothelial function. Hence, we examined their potential as biomarkers of sepsis.

**Methods:**

In total, 117 participants, including 20 healthy controls, 21 patients with systemic inflammatory response syndrome (SIRS), and 76 patients with sepsis, were enrolled in this study. Sepsis severity was assessed using the Acute Physiology and Chronic Health Evaluation (APACHE) II and the Sequential Organ Failure Assessment (SOFA) scores.

**Results:**

The Median plasma EphA2 levels increased progressively from healthy controls to SIRS and sepsis cases (154.29, 293.52, and 554.24 pg/mL; all *p* < 0.05). The median plasma Del-1 levels were highest in healthy controls, lowest in SIRS, and intermediate level in sepsis (101.27, 16.88, and 36.9 pg/mL; all *p* < 0.001). The levels of both biomarkers were higher in 28-day non-survivors than in survivors, in patients with sepsis (EphA2:898.09 vs. 475.88 pg/mL, *p* < 0.001; Del-1:46.09 vs. 32.68 pg/mL, *p* = 0.193); however, only EphA2 was statistically significant. The area under the curve for the EphA2 was 0.74 in the receiver operating characteristic curve analysis for predicting 28-day mortality, whereas APACHE II, SOFA, and Del-1 showed values of 0.762, 0.614, and 0.595, respectively. Kaplan–Meier analysis using these cutoffs revealed that survival was significantly higher in the group with both low EphA2 and Del-1 levels compared to the group with high levels of both markers (*p* < 0.001).

**Conclusion:**

Plasma EphA2 levels consistently increased with sepsis severity, suggesting its biomarker value for sepsis diagnosis and prognosis. In contrast, plasma Del-1 response was variable, indicating its limited prognostic utility.

## 1 Introduction

Sepsis is a critical health issue characterized by a dysregulated host response to infection that leads to life-threatening organ dysfunction (1). It is one of the leading causes of hospital mortality worldwide, with nearly 50 million cases of severe sepsis or septic shock annually and 11 million deaths worldwide (2). Furthermore, approximately half of all patients with sepsis are managed in the intensive care unit (ICU), and more than 25% of these patients die (3). Notably, vascular endothelial dysfunction is a key factor in the pathogenesis of sepsis. This dysfunction compromises the endothelial barrier and increases its permeability to fluids, proteins, and inflammatory cells (4–6), hence resulting in sepsis-associated organ failure and mortality (7, 8). Furthermore, recruitment and extravasation of leukocytes are involved in sepsis; however, these processes are essential for the immune response to infection or injury (9, 10). In addition, sepsis severity is exacerbated when leukocyte–endothelium interactions influence inflammation and endothelial damage (11, 12).

The erythropoietin-producing hepatocellular carcinoma (Eph)A2 belongs to the largest family of receptor tyrosine kinases (13) and significantly influences vascular formation and endothelial function. It interacts with the ephrin-A1 ligand and increases vascular permeability, contributing to lung injury. Vascular endothelial dysfunction is a critical factor in sepsis, and several studies suggest that EphA2 may influence inflammation by promoting endothelial injury (14, 15). Additionally, developmental endothelial locus-1 (Del-1, also known as EGF-like repeats and discoidin I-like domains 3, EDIL3) is a glycoprotein secreted by the endothelial cells, where it associates with the cell surface and extracellular matrix (16, 17). It helps in regulating inflammation, particularly by inhibiting leukocyte adhesion through antagonism of the lymphocyte function-associated antigen-1 (LFA-1) pathway, making it an endogenous inhibitor of leukocyte recruitment (10). Furthermore, it is expressed in various tissues, including the lungs, kidneys, and the central nervous system, where it participates in vascular homeostasis and angiogenesis (17). However, its anti-inflammatory properties contrast with the pro-inflammatory effects of the EphA2 during sepsis. This is because the EphA2 promotes vascular permeability and endothelial dysfunction contributing to sepsis progression; on the contrary, Del-1 suppresses excessive leukocyte recruitment and controls inflammation. The opposing roles of these receptors highlight the delicate balance between pro-inflammatory and anti-inflammatory mechanisms that underlie the pathophysiology of sepsis.

Therefore, in this study, we aimed to investigate how plasma EphA2 and Del-1, which are involved in vascular function and leukocyte migration, differ among healthy individuals, patients with systemic inflammatory response syndrome (SIRS), and patients with sepsis, focusing on their roles as sepsis biomarkers.

## 2 Materials and methods

### 2.1 Study participants

**Figure 1:**
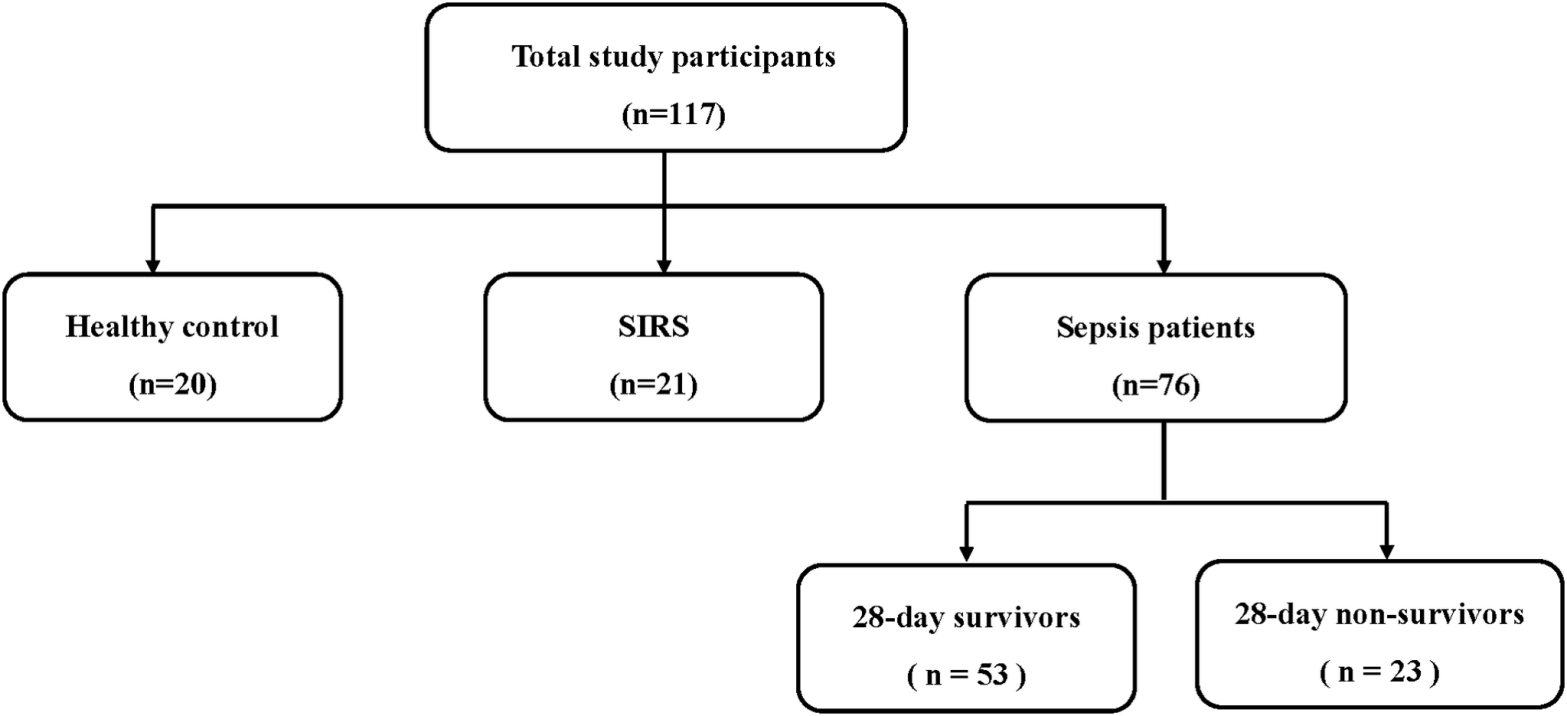
Flowchart of study participants.

Patients admitted to the ICU of Severance Hospital, a tertiary referral center in Seoul, South Korea, were prospectively enrolled between March 2017 and June 2018. The inclusion criteria were the presence of sepsis within the first 24 h of ICU admission, as defined by the Sepsis-3 criteria (infection + a Sequential Organ Failure Assessment (SOFA) score increase of ≥2) (1). All patients received standard sepsis treatment following the established guidelines (18). Subsequently, the patients were categorized as either survivors or non-survivors, based on their 28-day mortality (Figure 1). Furthermore, SIRS was defined when at least two of the following criteria were met: body temperature > 38°C or < 36°C, heart rate > 90 beats per minute, respiratory rate > 20 breaths per minute or PaCO_2_ < 32 mm Hg, and white blood cell count >12,000/μL, <4,000/μL, or > 10% immature forms. A total of 21 patients who did not meet the definition of sepsis but fulfilled the SIRS criteria were included. In addition, 20 healthy individuals who volunteered to participate in the study were included as controls. Patients with SIRS and healthy controls were recruited during the same period as the sepsis patients.

### 2.2 Data collection

Basic demographic information, including age, sex, and body mass index (BMI), was collected from all participating patients. Furthermore, the type of surgery performed was documented for patients with SIRS. However, additional data such as the Charlson Comorbidity Index, infection source, microbiological blood culture results, 28-day mortality, and laboratory findings were obtained from the hospital medical records for patients with sepsis. The severity of the patient’s conditions during the first 24 h following admission was assessed using the Acute Physiology and Chronic Health Evaluation II (APACHE II) and Sequential Organ Failure Assessment (SOFA) scoring systems.

### 2.3 Plasma EphA2 and Del-1 measurements

The human plasma used in this study was obtained through the following process. First, we collected plasma samples from healthy participants. Second, for plasma from patients with SIRS, we obtained the samples from postoperative orthopedic patients or SIRS patients admitted to the ICU. Third, we obtained plasma samples from adult patients with sepsis (>18 years old) admitted to the ICU of Severance Hospital for analysis. Furthermore, whole blood was collected from patients with sepsis, on the day of ICU admission (D0). Subsequently, plasma was prepared by centrifuging the whole blood for 15 min at 800×*g* and 4°C. The supernatants from centrifuged blood were immediately aliquoted and stored at −80°C until the analysis was performed. Further, plasma EphA2 levels were measured using a Human Magnetic Luminex Screening Assay Kit (R&D Systems Inc., Minneapolis, MN, United States). The kit detects all forms of the EphA2, including soluble, membrane-bound, phosphorylated, and non-phosphorylated forms. All samples and standards were assayed in duplicate using a Luminex 200TM System (Merck Millipore, Darmstadt, Germany). The plasma levels of DEL-1 (Human EDIL3) were measured using a commercially available enzyme-linked immunosorbent assay (ELISA) kit (Human EDIL3 ELISA, [R&D Systems™ DY604605]). The assay was performed following the manufacturer’s instructions.

### 2.4 Ethical approval

The study protocol was approved by the Institutional Review Board (IRB) of Severance Hospital (IRB no: 4-2017-0654). Written informed consent was obtained from patients or their guardians. All study procedures were performed following relevant guidelines and regulations.

### 2.5 Statistical analysis

Statistical analyses were performed using the R software (version 4.1.1; R Foundation for Statistical Computing, Vienna, Austria). Continuous variables are presented as mean ± standard deviation or median and interquartile range. In addition, continuous variables were compared using the Student’s t-test for parametric data or the Wilcoxon rank sum test for non-parametric data. Categorical variables are presented as numbers and percentages and were compared using the chi-square test or Fisher’s exact test. Furthermore, a non-parametric Kruskal–Wallis test was used to compare three or more groups for qualitative parameters. Data visualization, including boxplots showing the comparison of the EphA2 and Del-1 levels, was performed using the ggplot2 package. Area under the curve (AUC) analyses of the receiver operating characteristics (ROC) curves were performed to compare the plasma EphA2 levels, Del-1 levels, APACHE II scores, and SOFA scores. Based on the cutoffs derived from the ROC curves for EphA2 and Del-1 levels, patients were stratified into 4 groups, and Kaplan–Meier survival analyses were conducted. Statistical differences between survival curves were assessed using the log-rank test, with post-hoc pairwise comparisons adjusted by the Bonferroni correction to account for multiple comparisons. All tests were two-tailed, and statistical significance was set at *p* < 0.05.

## 3 Results

### 3.1 Study patients

In total, 117 participants were included in the study and divided into three groups: 20 healthy controls, 21 patients with SIRS, and 76 patients with sepsis. Fifty-three patients with sepsis were 28-day survivors, and 23 were 28-day non-survivors (Figure 1). The median age was significantly higher in the SIRS (59 years) and sepsis groups (70 years) than in the control group (38 years) (*p* < 0.001). However, no significant differences were found in BMI or sex between the groups. The SIRS group included eleven patients who underwent orthopedic surgery, three patients who underwent abdominal or liver surgery, two patients with exacerbation of interstitial lung disease (ILD), two patients with deterioration of liver disease, two patients with deterioration of acute kidney injury (AKI) or chronic kidney disease (CKD), and one patient who experienced drowning. Most of the patients with sepsis had pulmonary infections (40.8%), followed by urinary tract (28.9%), and abdominal infections (18.4%) (Table 1).

**Table 1:** Baseline characteristics of study participants.

Characteristic	Control (*n* = 20)	SIRS (*n* = 21)	Sepsis (*n* = 76)	*p*-value
Age (years), median (IQR)	38 (32–42.2)	59 (48–69)	70 (62–78)	<0.001
Gender, male, *n* (%)	8/20 (40)	15/21 (71.4)	46/76 (60.5)	0.11
BMI (kg/m^2^), median (IQR)	21.8 (20–23)	22.7 (21.2–24.4)	22.3 (20.3–25.6)	0.547
Post-operation patients, *n* (%)				
Ankle fracture operation		5 (23.8)		
DM foot amputation		2 (9.5)		
DM foot debridement		4 (19.0)		
Liver/abdomen		3 (14.3)		
ILD exacerbation		2 (9.5)		
Liver disease deterioration		2 (9.5)		
AKI/CKD deterioration		2 (9.5)		
Drowning		1 (4.8)		
Site of infection, *n* (%)				
Pulmonary			31 (40.8)	
Urinary tract			22 (28.9)	
Abdomen^a^			14 (18.4)	
Skin and soft tissue			4 (5.3)	
Others^b^			5 (6.6)	

Data are reported as medians (interquartile ranges [IQRs]) for continuous variables and numbers (percentages) for categorical variables. *p*-values obtained via Kruskal–Wallis test. SIRS, systemic inflammatory response syndrome. BMI, body mass index; DM, diabetes mellitus; ILD, interstitial lung disease; AKI, acute kidney injury; CKD, chronic kidney disease.

a Abdomen, gastrointestinal and hepatobiliary infections; peritonitis.

b Others: meningitis, spinal abscess, septic arthritis, primary unknown infection.

### 3.2 Differences in plasma EphA2 and Del-1 levels among healthy controls, SIRS, and sepsis patients

**Figure 2:**
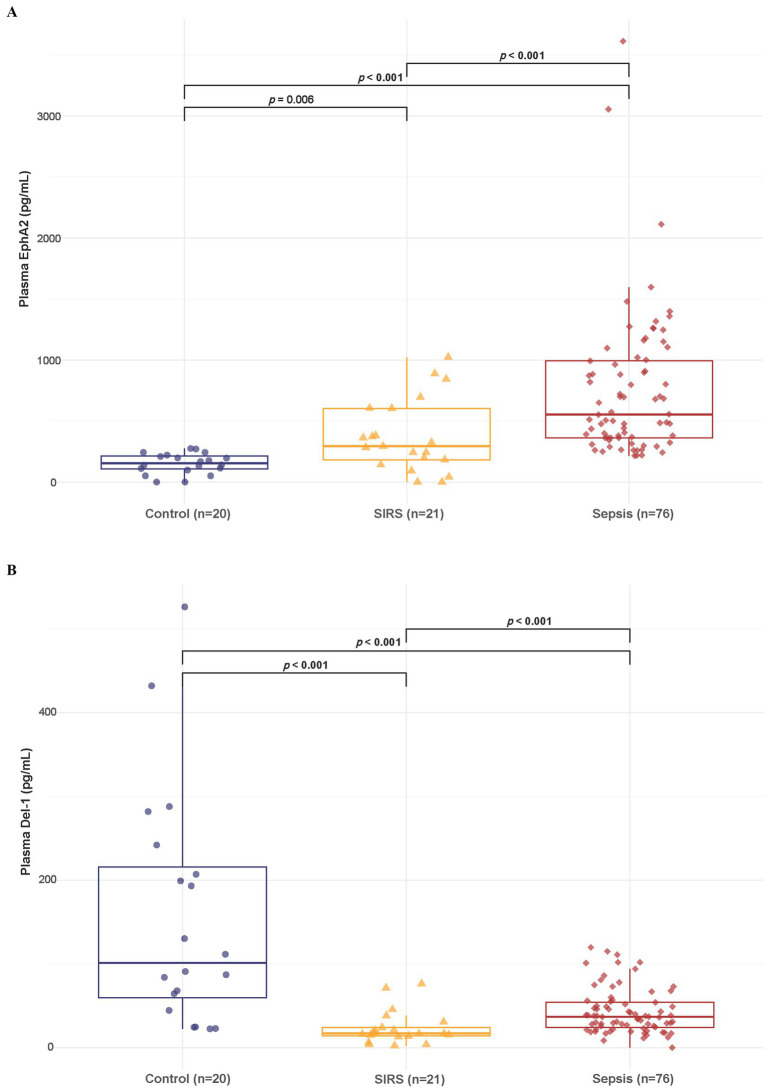
Differences in plasma EphA2 and Del-1 levels among healthy controls, SIRS, and patients with sepsis. **(A)** Boxplot shows that the median plasma EphA2 levels differed significantly across the three groups (Control: 154.29, SIRS: 293.52, Sepsis: 554.24 pg/mL, all *p* < 0.05). *p*-values obtained via Wilcoxon rank sum test. **(B)** Boxplot shows that the median plasma Del-1 levels were highest in healthy controls, lowest in SIRS, and intermediate level in sepsis (Control: 101.27, SIRS: 16.88, Sepsis: 36.9 pg/mL; all *p* < 0.001). *p*-values obtained via Wilcoxon rank sum test.

The box plot showed that the median plasma EphA2 levels differed significantly across the groups, with sepsis patients having notably higher levels than controls and SIRS (Control: 154.29, SIRS: 293.52, sepsis: 554.24 pg/mL, all *p* < 0.05) as shown in Figure 2A. Meanwhile, the median plasma Del-1 levels were highest in healthy controls, lowest in SIRS, and intermediate level in sepsis (Control: 101.27, SIRS: 16.88, sepsis: 36.9 pg/mL; all *p* < 0.001) (Figure 2B). Furthermore, when comparing plasma EphA2 and Del-1 levels among controls, SIRS, and sepsis patients within the 20–50 years age subgroup, consistent trends were observed (Supplementary Figure S1).

### 3.3 Differences in plasma EphA2 and Del-1 levels between 28-day survivors and non-survivors in sepsis patients

**Figure 3:**
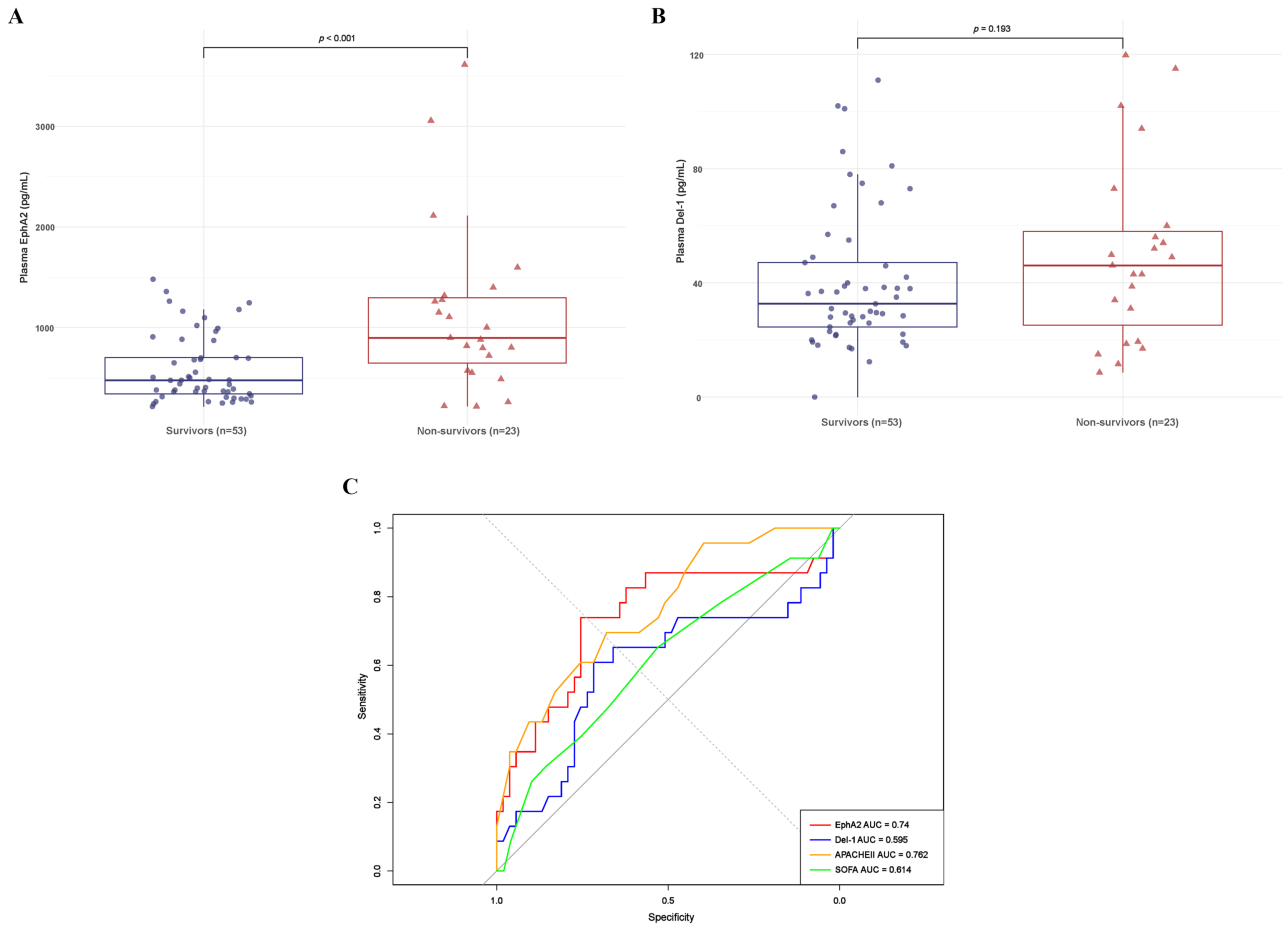
Differences in plasma EphA2 and Del-1 levels between 28-day survivors and non-survivors in patients with sepsis. **(A)** Boxplot shows the plasma EphA2 levels comparing 28-day survivors to non-survivors (median 475.88 vs. 898.09 pg/mL, p < 0.001). *p*-values obtained via Wilcoxon rank sum test. **(B)** Boxplot shows the plasma Del-1 levels comparing 28-day survivors to non-survivors (median 32.68 vs. 46.09 pg/mL, *p* = 0.193). *p*-values obtained via Wilcoxon rank sum test. **(C)** ROC curve for predicting 28-day mortality using EphA2, Del-1, APACHE II, and SOFA scores.

Table 2 shows the differences in clinical and laboratory parameters according to the 28-day mortality. Non-survivors had a significantly higher Charlson Comorbidity Index and a higher rate of continuous renal replacement therapy than survivors. In addition, the APACHE II score significantly differed among the severity scores for non-survivors compared with survivors. Furthermore, non-survivors showed significantly higher median levels of plasma EphA2 than survivors, regarding EphA2 levels (898.09 vs. 475.88 pg/mL, *p* < 0.001, Figure 3A), indicating that elevated EphA2 levels may be associated with worse outcomes in sepsis. In addition, median plasma Del-1 levels were also higher in non-survivors than in survivors; however, the difference was not statistically significant (46.09 vs. 32.68 pg/mL, *p* = 0.193, Figure 3B). The ROC curve for 28-day mortality analysis shows that EphA2 (AUC = 0.74) demonstrates a moderate level of performance, comparable to APACHE II (AUC = 0.762), while Del-1 (AUC = 0.595) exhibits weaker predictive performance, falling below the threshold typically considered acceptable for diagnostic utility (Figure 3C).

**Table 2:** Comparison of characteristics of patients with sepsis according to 28-day mortality.

Characteristic	28-day survivor (*n* = 53)	28-day non-survivors (*n* = 23)	*p*-value
Age (years), median (IQR)	70.0 (62–77)	70.0 (66–78.5)	0.519
Gender, male, *n* (%)	34/53 (64.2)	12/23 (52.2)	0.468
BMI (kg/m^2^)	22.5 (21.3–25.4)	21.9 (19.2–25.6)	0.288
Site of infection, *n* (%)			
Pulmonary	20 (37.7)	11 (47.8)	
Urinary tract	21 (39.6)	1 (4.3)	
Abdomen^a^	6 (11.3)	8 (34.8)	0.015
Skin and soft tissue	3 (5.7)	1 (4.3)	
Others^b^	3 (5.7)	2 (8.7)	
Charlson comorbidity index	5 (3–6)	6 (4–8)	0.017
Clinical parameters, *n* (%)			
Positive blood culture	24 (45.3)	9 (39.1)	0.806
CRRT	18 (33.9)	17 (73.9)	0.005
Mechanical ventilation	17 (32.1)	13 (56.5)	0.081
Clinical severity score			
APACHE II score	21 (15–29)	32 (22.5–39)	<0.001
SOFA score	8 (7–10)	9 (8–12.5)	0.120
Laboratory parameters			
Leukocytes (× 10^6^/mL)	12.74 (7.96–17.15)	14.41 (6.51–19.89)	0.779
Platelets (× 10^6^/mL)	121 (80–187)	172.5 (72.25–222)	0.629
CRP (mg/L)	236.5 (131.9–298.8)	164 (123.7–224.58)	0.082
Procalcitonin (ng/mL)	25.22 (3.58–93)	17.9 (2.11–35.67)	0.216
Lactate (mmol/L)	2.6 (2–3.9)	2.6 (1.75–8.6)	0.311
Albumin (g/dL)	2.6 (2.1–2.8)	2.3 (1.95–2.58)	0.132
Total Bilirubin (mg/dL)	0.7 (0.4–1.2)	0.6 (0.33–1.15)	0.931
BUN (mg/dL)	38 (21.8–66.1)	36.4 (26–61.3)	0.870
Creatinine (mg/dL)	2.12 (1.25–3.15)	2.65 (1.67–3.89)	0.493
Plasma EphA2 (pg/mL)	475.88 (342.45–702.03)	898.09 (647.15–1295.67)	<0.001
Plasma Del-1 (pg/mL)	32.68 (24.57–47.11)	46.09 (25.19–58)	0.193

Data are reported as medians (interquartile ranges [IQRs]) for continuous variables and numbers (percentages) for categorical variables. SIRS, systemic inflammatory response syndrome. BMI, body mass index; CRRT, continuous renal replacement therapy; APACHE II, Acute Physiology and Chronic Health Evaluation II; SOFA, Sequential Organ Failure Assessment; EphA2, erythropoietin-producing hepatocellular carcinoma A2; Del-1, developmental endothelial locus-1.

a Abdomen: gastrointestinal and hepatobiliary infections, peritonitis.

b Others: meningitis, spinal abscess, septic arthritis, primary unknown infection.

### 3.4 Impact of plasma EphA2 and Del-1 levels on 28-day survival in sepsis patients

**Figure 4:**
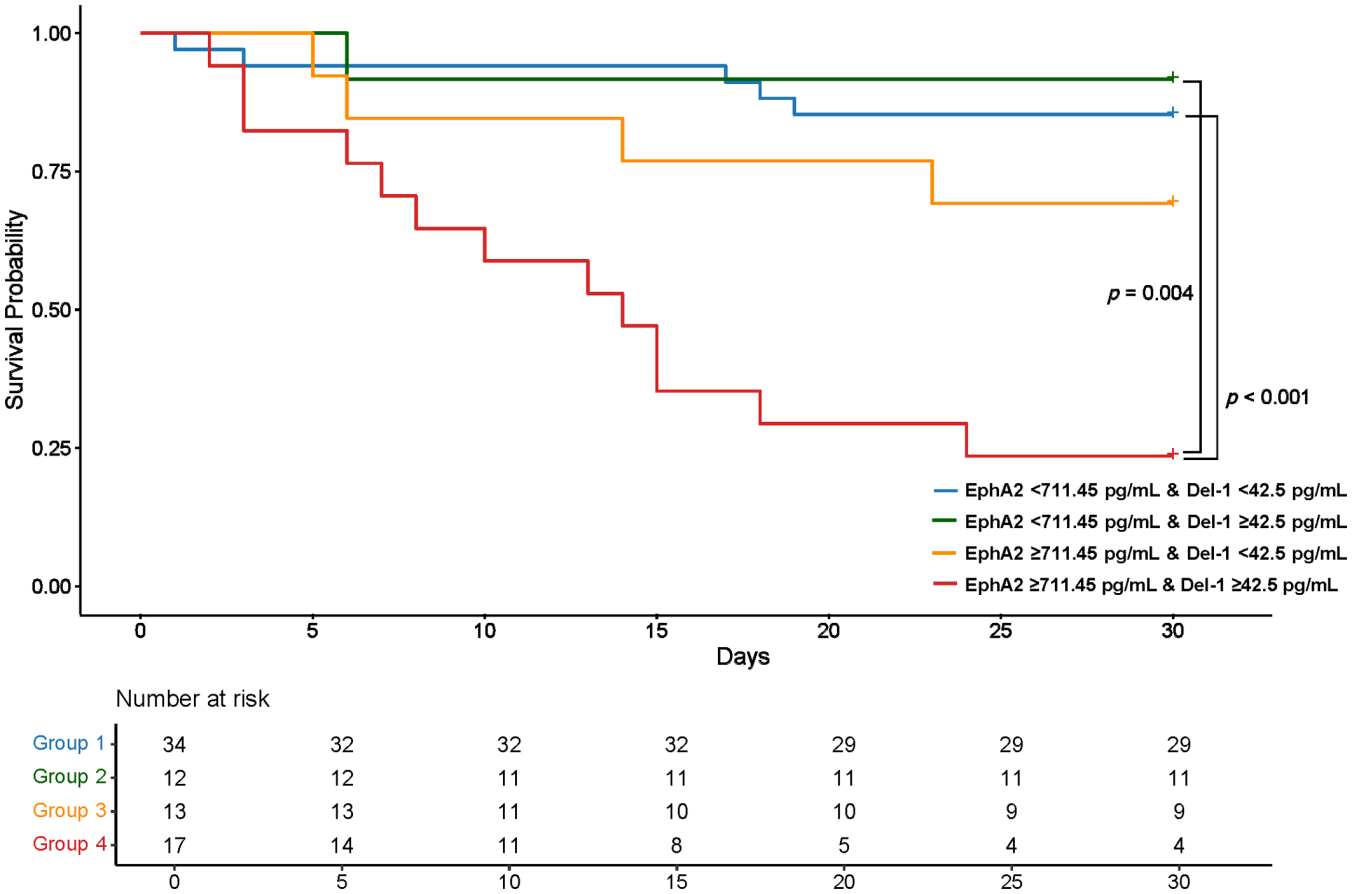
Kaplan–Meier Survival Curves Based on plasma EphA2 and Del-1 Cutoffs Kaplan–Meier survival curves stratified by four groups according to EphA2 (711.45 pg/mL) and Del-1 (42.5 pg/mL) cutoffs. Group 1, with both markers below their respective cutoffs, showed significantly higher survival compared to Group 4, where both markers exceeded the cutoffs (*p* < 0.001). Group 2, with low EphA2 and high Del-1, also exhibited significantly better survival than Group 4 (*p* = 0.004). No statistically significant differences were observed between the other groups. *p*-values were calculated using the log-rank test, with post-hoc pairwise comparisons adjusted by the Bonferroni correction.

Using the EphA2 cutoff of 711.45 pg/mL and the Del-1 cutoff of 42.5 pg/mL, determined from the ROC curve in Figure 3C for mortality prediction, four groups were established to assess Kaplan–Meier survival (Figure 4). Group 1, where both EphA2 and Del-1 levels were below their respective cutoffs (blue line), exhibited significantly higher survival compared to Group 4, where both markers were above the cutoffs (red line) (*p* < 0.001). Group 2, where EphA2 levels were below the cutoff and Del-1 levels exceeded the cutoff (green line), also demonstrated significantly improved survival relative to Group 4 (*p* = 0.004). No statistically significant differences were observed between the other groups.

### 3.5 Relative changes in EphA2 and Del-1 levels across control, SIRS, and sepsis groups according to disease severity

**Figure 5:**
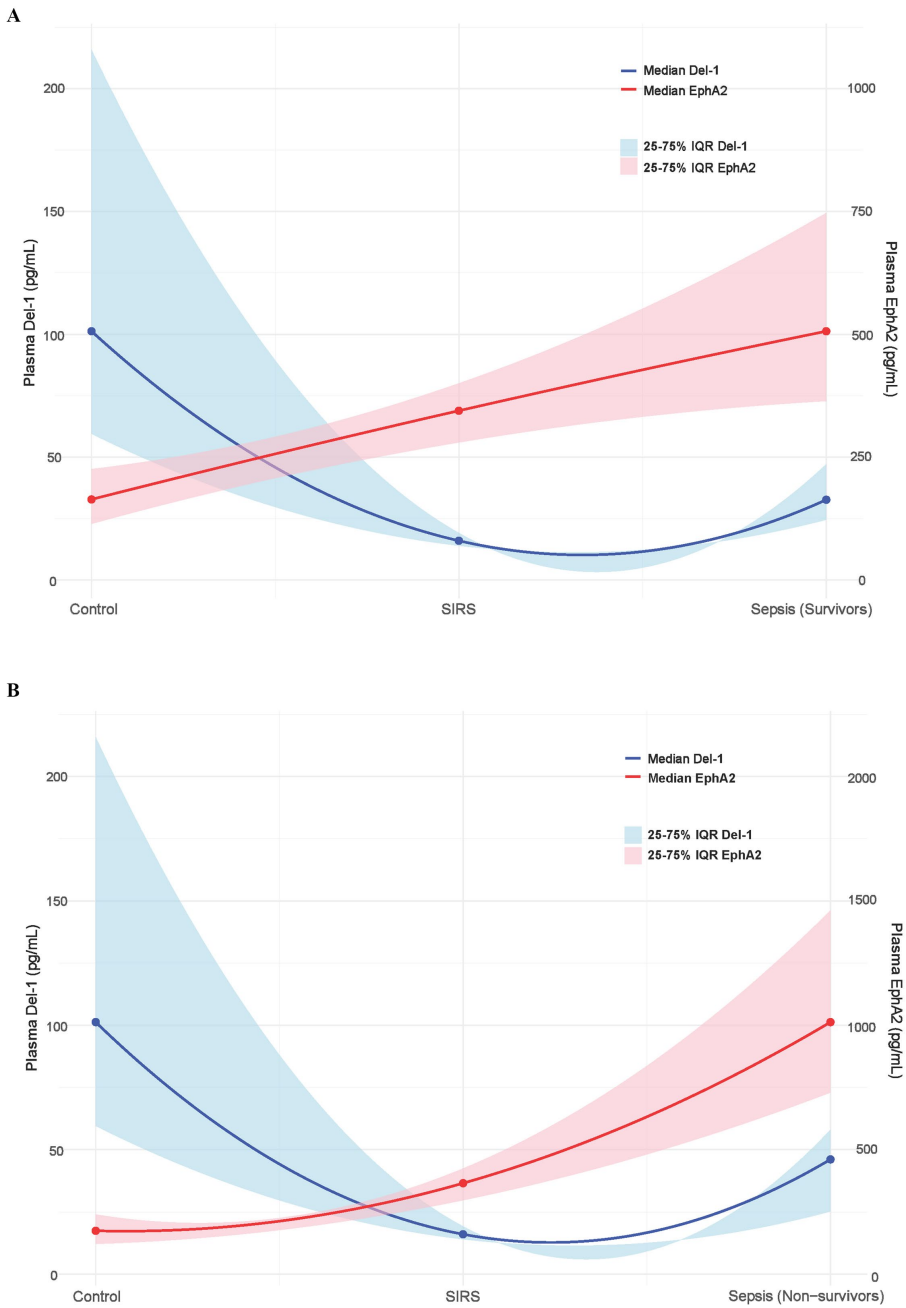
Relative changes in plasma EphA2 and Del-1 levels across control, SIRS, and survivors and non-survivors of sepsis. The blue solid line represents the median Del-1 value, and the blue shaded area represents the 25–75% interquartile range (IQR) of Del-1. The solid red line shows the median EphA2 values, whereas the shaded red area represents the 25–75% IQR of EphA2. **(A)** Control, SIRS, sepsis survivors. **(B)** Control, SIRS, sepsis non-survivors.

Figure 5 shows how the plasma EphA2 (red line) and Del-1 (blue line) levels differed across healthy controls, patients with SIRS, sepsis survivors (Figure 5A), and sepsis non-survivors groups (Figure 5B). The plasma EphA2 levels increased progressively from the controls to patients with SIRS and sepsis, with the highest levels observed in sepsis non-survivors, indicating an association with worsening prognosis. In contrast, plasma Del-1 levels were the lowest in SIRS patients compared with healthy controls; however, the levels tended to increase as sepsis progressed, when compared with those in patients with SIRS (Figures 5A,B).

## 4 Discussion

In this study, we compared the plasma levels of the EphA2 and Del-1 among healthy controls, patients with SIRS, and patients with sepsis. Our findings showed that plasma EphA2 levels progressively increased from controls to patients with SIRS and sepsis, with higher levels corresponding to greater sepsis severity. In contrast, plasma Del-1 levels were lowest in SIRS patients compared with controls; however, it showed an increasing trend in sepsis.

EphA2 plays a critical role in sepsis by mediating inflammation and endothelial dysfunction (19). Previous studies have shown that EphA2 activation contributes to vascular permeability and leukocyte migration, both of which are key processes in the pathophysiology of sepsis (15, 19). Furthermore, elevated EphA2 levels are associated with poor outcomes in severe sepsis, possibly because they influence endothelial barrier breakdown and exacerbate systemic inflammation in severe sepsis (20). Consistent with these findings, our study showed that plasma EphA2 levels progressively increased with sepsis severity, showing significantly higher levels in patients with sepsis than in healthy controls and patients with SIRS. This consistent pattern reinforces previous research indicating that EphA2 is closely associated with sepsis progression and could serve as a potential biomarker for both the diagnosis and prognosis of patients with sepsis.

On the other hand, plasma Del-1 levels decreased to their lowest point in SIRS patients compared with healthy controls; however, showed a tendency to increase as sepsis progressed. This phenomenon may have resulted from several factors. First, during the early stages of SIRS, pro-inflammatory cytokines such as TNF-*α* and IL-17A are upregulated, suppressing the expression of Del-1. This suppression allows for a robust inflammatory response, facilitating neutrophil migration to inflamed tissues and enhancing the immune system’s ability to combat initial insults (21, 22). This mechanism aligns with the homeostatic need to promote a strong inflammatory response during the initial phase of SIRS (17, 23). Second, as inflammation progresses to sepsis, compensatory anti-inflammatory mechanisms, including the release of IL-10, are activated to counterbalance excessive inflammation. IL-10 has been shown to upregulate Del-1 expression, thereby reversing the suppressive effects of pro-inflammatory cytokines like IL-17A (22). This dynamic shift reflects the body’s effort to restore immune balance and prevent tissue damage caused by prolonged inflammation. Furthermore, this increases in Del-1 levels during sepsis highlights the intricate feedback regulation between immune activation and suppression, particularly in severe cases where immune dysregulation occurs. Del-1’s role in inhibiting neutrophil migration further supports this balance by limiting excessive immune cell infiltration and promoting resolution of inflammation (24, 25). These findings emphasize the complex interplay between cytokines and Del-1 in maintaining immune homeostasis under inflammatory and septic conditions (22, 26).

Notably in this study, we observed changes in plasma EphA2 and Del-1 levels in patients with sepsis, across healthy controls, and SIRS patients. These changes also varied with sepsis severity. The approach highlights how these two biomarkers, with contrasting roles, show dynamic changes in response to normal conditions, inflammation, and sepsis. In particular, this study underscores that Del-1 is involved in modulating inflammation; however, its levels neither continuously increase nor decrease, offering insights into the complex regulatory mechanisms of the immune response. There are numerous ongoing efforts to identify biomarkers for the early detection and severity prediction of sepsis. Some of these approaches include metabolomics, proteomics, and transcriptomics (27–29). However, as demonstrated in this study, it is crucial to assess not only patients with sepsis but also healthy controls and non-septic inflammatory patients (such as SIRS), as well as patients across different levels of sepsis severity. This comprehensive assessment can provide deeper insights into complex immune mechanisms and immune regulation during sepsis.

However, the single-center design, small sample size, significant age and comorbidity differences between healthy controls and patients are limitations of this study. This makes it difficult to generalize our findings. Additionally, the study was limited to one-time measurements of biomarker levels in sepsis patients, without incorporating serial measurements over time, restricting its ability to fully capture the dynamic course of the disease. Nevertheless, the study findings provide valuable insights into the relative changes in plasma EphA2 and Del-1 under different conditions, contributing to our understanding of the immune response in various health and disease states.

In conclusion, for the diagnosis and severity of sepsis, plasma EphA2, which shows a consistent upward trend as the disease progresses, had a greater diagnostic value than plasma Del-1, which exhibits varying changes throughout the disease. Further multicenter studies with larger, more balanced cohorts are essential to validate our findings and provide deeper insights into these complex regulatory mechanisms.

## Data Availability

The original contributions presented in the study are included in the article/Supplementary material, further inquiries can be directed to the corresponding author.
