# Supplementary material for: Plasma EphA2 level is a superior biomarker to Del-1 for sepsis diagnosis and prognosis
Source: Front Med (Lausanne). 2025 Jan 24;12:1505882. doi: 10.3389/fmed.2025.1505882 (PMC11802418; doi:10.3389/fmed.2025.1505882)
Supplement: Supplementary file 1 [file Supplementary_file_1.docx]

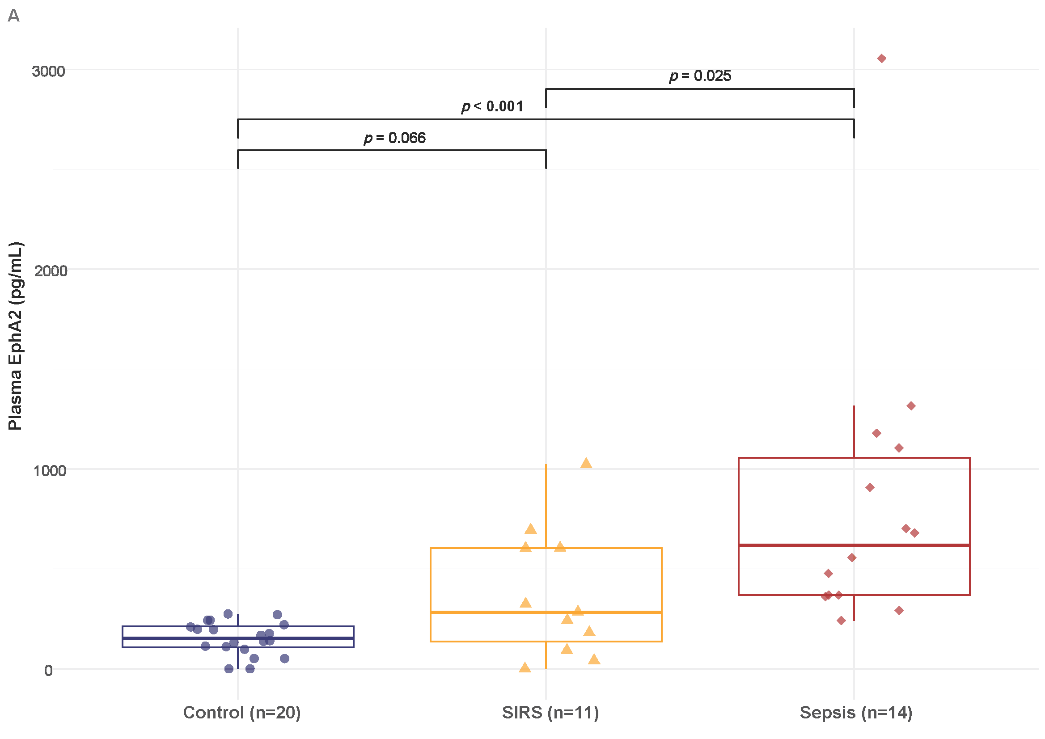

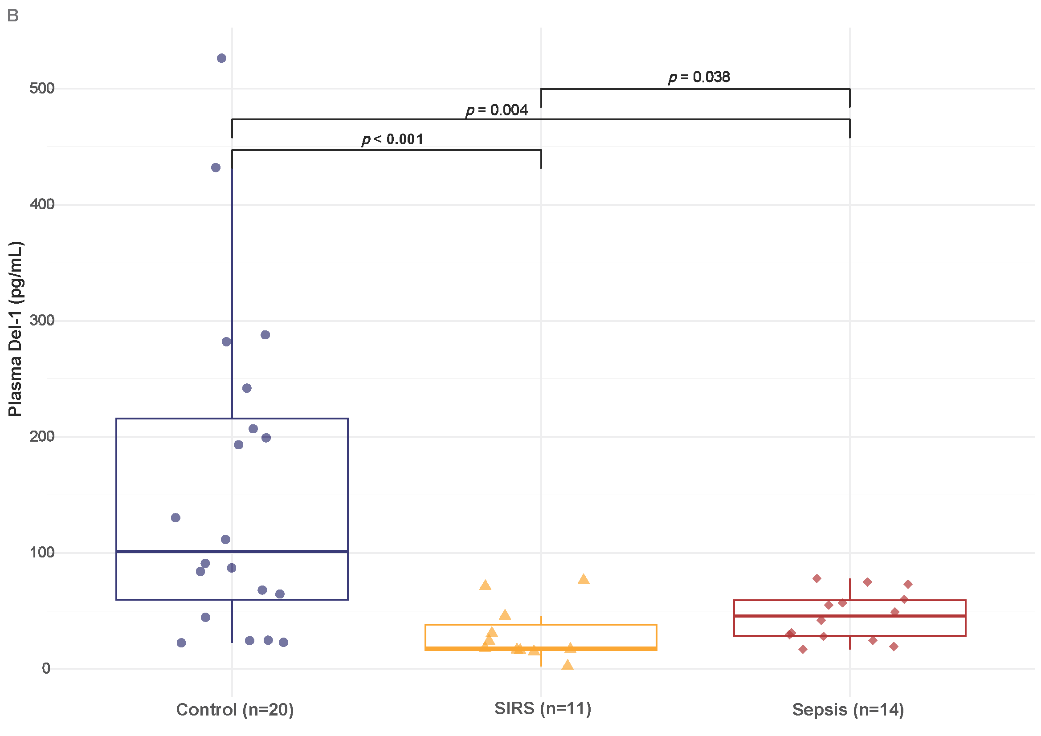


**Supplementary Figure S1. Plasma EphA2 and Del-1 levels in healthy controls, SIRS, and sepsis patients aged 20–50 years.**

(A) Boxplot shows that the median plasma EphA2 levels varied among the three groups aged 20-50 years (Control: 154.29 pg/mL, SIRS: 283.95 pg/mL, Sepsis: 618.28 pg/mL). Pairwise comparisons revealed the following p-values: Control vs. SIRS: p = 0.066, SIRS vs. Sepsis: p = 0.025, Control vs. Sepsis: p < 0.001 (Wilcoxon rank sum test). (B) Boxplot shows that the median plasma Del-1 levels were highest in healthy controls, lowest in SIRS, and intermediate level in sepsis (Control: 101.27 pg/mL, SIRS: 18 pg/mL, Sepsis: 45.5 pg/mL; all p < 0.05). p-values obtained via Wilcoxon rank sum test.
